# Systemic treatments for hepatocellular carcinoma: challenges and future perspectives

**DOI:** 10.2217/hep-2017-0020

**Published:** 2018-02-08

**Authors:** Francesco Tovoli, Giulia Negrini, Francesca Benevento, Chiara Faggiano, Elisabetta Goio, Alessandro Granito

**Affiliations:** 1 Department of Medical & Surgical Sciences, University of Bologna, S Orsola-Malpighi Hospital, Bologna, Italy

**Keywords:** cabozantinib, hepatocellular carcinoma, immunotherapy, lenvatinib, metronomic capecitabine, regorafenib, sorafenib, systemic therapies

## Abstract

Sorafenib has been the only approved systemic treatment of hepatocellular carcinoma (HCC) for almost a decade. Recently, two new drugs showed positive results in two Phase III studies. The RESORCE trial identified regorafenib as a valid second-line treatment for patients progressing to sorafenib, the REFLECT trial showed that lenvatinib is noninferior to sorafenib as front-line treatment. Following these trials, the therapeutic scenario will be dominated by anti-VEGFR drugs, with three different molecules showing a proven anticancer activity. Some open problems still remain and different immunotherapy trials are underway, following promising preliminary results. In this review we analyze: the most recent advancements about patients treated with sorafenib; the results of RESORCE and REFLECT trials; and the ongoing Phase III clinical trials. Finally, we discuss how they could address the current problems and possibly reshape the future of the systemic treatments for HCC.

The identification of effective systemic treatments for hepatocellular carcinoma (HCC) has always been a difficult challenge in oncology. Conventional chemotherapies, including doxorubicin and gemcitabine, failed to improve the overall survival (OS) of these patients [1]. Only in 2008, sorafenib, a VEGFR-inhibitor, demonstrated its efficacy in treating unresectable HCC in two randomized-controlled trials (RCTs) [2,3].

These results were followed by a period of enthusiasm in which the difficulties of the past seemed to be overcome. This period was soon dissipated by the failure of numerous RCTs with the consequent inability to find either a second-line treatment or a therapeutic alternative for patients intolerant to sorafenib (Table 1) [4–10].

**Table 1.** **Failed Phase III randomized clinical trials investigating drugs for patients with unresectable hepatocellular carcinoma not amenable for locoregional treatments.**

**Name of the trial**	**Study drug**	**Therapeutic line**	**Primary end point**
No name	Linifanib vs sorafenib	1	No difference in OS (HR: 1.046; 95% CI: 0.896–1.221)

SEARCH	Sorafenib + erlotinib vs sorafenib + placebo	1	No difference in OS (HR: 0.929; 95% CI: 0.781–1.106; p = 0.408)

No name	Sunitinib vs sorafenib	1	Significant inferiority in OS (HR: 1.30; 95% CI: 1.13–1.50; p = 0.014)

BRISK-FL	Brivanib vs sorafenib	1	No difference in OS (HR: 1.06; 95% CI: 0.93–1.22)

BRISK-PS	Brivanib vs placebo	2	No difference in OS (HR: 0.89; 95% CI: 0.69–1.15; p = 0.33)

REACH	Ramucirumab vs placebo	2	No difference in OS (HR: 0.87; 95% CI: 0.72–1.05; p = 0.14)

EVOLVE-1	Everolimus vs placebo	2	No difference in OS (HR: 1.05; 95% CI: 0.86–1.27; p = 0.68)

POLARIS2009-001	ADI-PEG20 vs placebo	2	No difference in OS (HR: 1.02; 95% CI: 0.85–1.23; p = 0.88)

METIV-HCC	Tivantinib vs placebo	2	No difference in OS (HR: 0.97; 95% CI: 0.75–1.25; p = 0.81)

JET-HCC	Tivantinib vs placebo	2	No difference in PFS (further data still not released)

HR: Hazard ratio; OS: Overall survival; PFS: Progression-free survival.

After almost a decade of disappointing results, new positive results arrived in 2016 and 2017. Regorafenib, another VEGFR-inhibitor demonstrated its efficacy as a second-line treatment [11]. In the front-line setting, a noninferiority Phase III RCT comparing lenvatinib versus sorafenib reached its primary end point [12]. Besides, the encouraging preliminary results of immunotherapy trials seem to authorize a cautious enthusiasm for the possible novel and highly innovative treatments [13,14].

The aims of this review are: to summarize the most important studies concerning sorafenib treatment published in the last years; critically analyze the results of the successful trial of the last year (regorafenib and lenvatinib); report ongoing Phase III studies, speculating on possible therapeutic innovations.

## Sorafenib & next-to-be-approved treatments

### Sorafenib

To date, sorafenib is the only systemic agent globally available for use in HCC. It is an orally active multitarget kinase inhibitor targeting different cell surface tyrosine kinases (e.g., VEGFR-1, -2, -3 and PDGFR-β) as well as the intracellular serine-threonine kinases Raf-1 and B-Raf. As such, sorafenib inhibits tumor cell proliferation and angiogenesis and promotes tumor cell apoptosis [15–17].

The antitumor activity of sorafenib in HCC was assessed in two large double-blind, placebo-controlled, Phase III RCTs: the SHARP and the Asia-Pacific trials [2,3]. Both trials demonstrated that sorafenib at the dose of 400 mg twice-daily significantly improved OS in patients with HCC not amenable for loco-regional procedures, well-preserved liver function (97% Child-Pugh A) and Eastern Co-operative Oncology Group (ECOG) performance status ≤2 (Table 2). Subsequent sub analyses of the SHARP trial showed that sorafenib was effective for the treatment of HCC regardless of HCC etiology, tumor burden, tumor stage (BCLC-B or C), performance status (Eastern Co-operative Oncology Group 0, 1 or 2) and prior treatment (curative therapy or chemoembolization) [18–20]. However, the survival benefit is greater in patients with disease confined to the liver (without extrahepatic spread), or in those with hepatitis C virus, or a lower neutrophil-to-lymphocyte ratio, an indicator of inflammation status, as recently demonstrated in a pooled exploratory analysis of the two registrative Phase III studies [19].

**Table 2.** **Rate of dose reduction and permanent drug discontinuation due to adverse events among patients treated with sorafenib for hepatocellular carcinoma in randomized clinical trials and in observational studies.**

**Study**	**Dose reduction (%)**	**Treatment discontinuation (%)**
SHARP	26	38

Asia-Pacific	30.9	19.5

SOFIA	54	44

GIDEON Child-Pugh A	40	29

GIDEON Child-Pugh B	29	40

INSIGHT	58.6	15.5

A series of real-line clinical trials performed in different geographical regions confirmed these favorable data [21–23]. In particular, the results of the GIDEON study [21], the largest real-life observational study involving 3371 patients treated with sorafenib across 39 different countries, confirmed a good efficacy and safety profile. Consequently, all of the current international guidelines unanimously recognize sorafenib as the standard treatment for Child-Pugh A patients who have been diagnosed with advanced HCC [24].

The benefit of sorafenib in the Child-Pugh B population, for which the underlying cirrhosis represent a competitive cause of death, is highly debated. Because sorafenib is metabolized primarily in the liver by cytochrome P450 3A4 and UGT1A9, a more compromised hepatic function might alter drug clearance and dose-related toxicity [25,26]. In a preceding Phase II trial enrolling 137 patients, 38 (28%) were classified as CPT B. The drug-related toxicity was similar between Child-Pugh A and Child-Pugh B patients however, the latter patients had a higher frequency of hyperbilirubinaemia (40 vs 18%), encephalopathy (11 vs 2%) and severe ascites (18 vs 11%).

A comparison of the pharmacokinetic parameters showed no significant differences between Child-Pugh A and Child-Pugh B patients. Although this finding was then confirmed by a Japanese Phase I study, the patient numbers of these two studies were too small to allow a meaningful pharmacokinetic comparison between the Child-Pugh classes [26].

In the two registrative trials were almost exclusively included Child-pugh A patients, with only 3–5% classed as CPT B patients [2,3].

As such most evidences rely on real-life studies showing a rate of adverse effects in Child-Pugh B patients quite similar to that of Child-Pugh A counterpart, but only a narrow survival benefit [21–23,27–29]. Consequently, some international guidelines (Asia-Pacific Association for the Study of the Liver, National Comprehensive Cancer Network and European Association for the Study of the Liver) suggest a possible use of sorafenib even in Child-Pugh B patients, but limited to patients with a Child-Pugh score of 7 with mild elevation of bilirubin serum concentration and without diuretic uncontrolled ascites. However, Child-Pugh B, as well as elderly patients, represents a fragile population more often requiring a reduced dosage and a stricter monitoring [30–32].

Sorafenib is not free from adverse effects (the most frequent being skin toxicity, diarrhea, arterial hypertension and fatigue), potentially leading to dose reduction or treatment discontinuation [2–3,30]. Interestingly, data from field-practice studies (SOFIA, INSIGHT and GIDEON studies) point out a little greater rate of dose reduction or treatment discontinuation due to adverse events, reflecting the presence of comorbid patients in the real world (Table 2) [21–23].

In the GIDEON study the overall frequency of adverse effects (84 vs 89%) and those considered to be drug-related adverse events (69 vs 64%) was generally consistent across the Child-Pugh A and Child-Pugh B patients, as well as the frequency of grade 3 and 4 adverse events. However, serious adverse events (60 vs 36%) and treatment discontinuation (40 vs 29%) due to adverse events were more common in Child-Pugh B patients than in Child-Pugh A patients. The incidence of individual adverse events and drug-related adverse events did not differ between Child-Pugh A and Child-Pugh B patients, with the exception of hand–foot skin reaction, which occurred more frequently in Child-Pugh A patients (32 vs 17%) [21].

In this setting, a topic of growing interest is the prognostic significance of adverse events. As a matter of fact, at the moment there is a lack of biomarkers or other plasma predictors of sorafenib efficacy [33,34]. As many studies have pointed out, HCC has a significant genetic heterogeneity that can lead to primary or acquired resistance to sorafenib [35]. Although several mechanisms involved in this process have been recognized, we are far from identifying reliable biomarkers predictive of sorafenib resistance [36]. Hence, research has looked for other markers such as clinical predictors. Indeed, some studies tried to assess the correlation between adverse event development and treatment outcome [37–41], based on the concept that the occurrence of adverse events may be related to the sorafenib mechanism of action inhibiting one or more drug molecular targets. Early dermatologic adverse events currently appear to have a more convincing predictor role, since in a prospective study their appearance was significantly correlated with longer OS and time to progression (TTP) compared with patients not experiencing these symptoms, and resulted also correlated with complete response [39,40]. These studies call for research to validate this prognostic correlation, but they also pose further questions on the optimal management of patients without these specific side effects to improve the cost–effectiveness of sorafenib [42].

It is also worth of note that a significant interpatient variability of sorafenib pharmacokinetic has been reported and the available data would suggest a relationship between increased cumulated sorafenib exposure and adverse events [43]. An additional source of inter-individual different toxicity profile could be related to the genetic polymorphisms in VEGF and VEGFR [44].

Immediately after its approval as front-line systemic treatment, different studies verified the suitability of sorafenib as adjuvant therapy. In particular the Phase III STORM trial enrolled 1114 patients treated with liver resection or percutenous ablation. Patients were randomized in a 1:1 fashion and received either sorafenib or placebo. The main end point was recurrence-free survival, which was found to be similar in the two groups (33.3 months in the sorafenib group vs 33.7 months in the placebo group; hazard ratio [HR]: 0.940; 95% CI: 0.780–1.134; p = 0.26) [45].

Besides, a combination of locoregional and systemic treatments (chemoembolization + sorafenib) in patients with intermediate-stage HCC has been largely investigated in recent years [46–48]. Overall, the results suggest that this combination failed to result in meaningful benefits, showing longer time to progression, but without improving the OS [49].

Further, the results of a Phase I trial strongly discouraged a combined strategy of Stereotactic Body Radiation Therapy and concurrent use of sorafenib due to the high prevalence of severe adverse events, including two cases of ruptured HCC [50].

Currently, both the STOP-HCC trial [51] and a specifically dedicated cohort of the SORAMIC trial [52] are evaluating whether a combination of TARE and sorafenib may prolong the OS of patients with locally advanced HCC compared with sorafenib alone.

In absence of second-line treatments approved, the question whether sorafenib should be stopped on radiologic progression alone still remains a clinically relevant topic. In the SHARP trial the authors made clear that the statistical significance was obtained also thanks to the possibility to treat patients even beyond radiologic progression, provided that the investigator believed it was beneficial for the patient [2].

The benefit of treatment beyond radiological progression was confirmed by real-life studies, which also demonstrated that a tumor burst may occur at the withdrawal of sorafenib. The reasons for this rapid tumor spread are complex but probably involve the reactivation of the previously inhibited VEGFR pathway, together with the acquired activation of alternative pathways [53]. As such, while waiting the approval of second-line therapies for HCC progressors, sorafenib could still be offered beyond radiological, but not symptomatic progression. Once second-line treatments will be available, the most appropriate management after sorafenib failure will have to be investigated in future large trials [54].

The availability of regorafenib as a second-line therapy may modify this orientation and will lead to future studies to identify the best timing for a therapy switch.

Some information about this topic may be already deducted from two important studies. Reig *et al*. [55] described prospectively followed 147 HCC patients treated with sorafenib. Radiological response was assessed after 1 month and then every 8 weeks. Baseline BCLC C status, deterioration of liver function, compromised performance status, time to progression and permanent sorafenib withdrawal were all independently associated with a shortened OS. More interestingly, the authors described four different pattern of progression (intrahepatic increase in tumor size, extrahepatic increase in tumor size, new intrahepatic lesions, new extrahepatic lesions) and found that the latter pattern is also independently associated with a worse OS. As such, the authors concluded that not all pattern of progression are equal and that the BCLC classification should be refined at the time of progression to better predict the prognosis. Iavarone *et al*. analyzed 200 patients who permanently discontinued sorafenib and were potentially eligible for second-line treatments [56]. Performance status, macrovascular invasion and extrahepatic spread were shown to be prognosticators of OS, but the most important finding was that the reason for sorafenib discontinuation was an independent prognostic factor as well. In particular, discontinuation due to adverse effects was associated with the best prognosis.

The findings by Iavarone and Reig brought important considerations which are useful in clinical practice and also represent critical factors in the design of clinical trials [57].

Finally, it has been recently reported that some scores mainly based on biochemical parameters, could be useful to predict the clinical benefit of sorafenib treatment as well as to better select patients for second-line treatment after sorafenib failure [58–60]. However their use in clinical practice need validations studies.

### Lenvatinib

Lenvatinib is an oral multikinase inhibitor targeting the VEGFR, FGFR and PDGFR, as well as the RET and KIT pathways [61]. As such, its biological action is similar but completely overlapping with that of sorafenib. Currently, lenvatinib is approved in differentiated thyroid cancers refractory to radio-iodine administration [62]. Further, a combination of lenvatinib and everolimus has been approved as a second-line treatment for advanced renal cell carcinoma [63].

The efficacy and the safety of lenvatinib as a front-line treatment for advanced HCC has been tested in the recently concluded REFLECT trial. This non-inferiority Phase III trial randomized 954 patients with unresectable HCC to lenvatinib (8 mg or 12 mg once-daily based on body weight) or sorafenib at 400 mg twice-daily [12].

The primary end point of a noninferior OS was met (13.6 vs 12.3 months; HR: 0.92; 95% CI: 0.79–1.06), making REFLECT the first positive global Phase III trial compared with sorafenib in first-line treatment for HCC. The median time to progression was 8.9 months with lenvatinib and 3.7 months with sorafenib (HR: 0.63; p < 0.00001). In addition, lenvatinib demonstrated a significantly higher overall response rate compared with sorafenib (24% vs 9%; odds ratio: 3.13; p < 0.00001). A similar number of patients in both arms had treatment-emergent adverse events. Most common treatment-emergent adverse events in the lenvatinib arm were hypertension (42%), diarrhea (39%), decreased appetite (34%), decreased weight (31%) and fatigue (30%). Median treatment duration was 5.7 months (0–35) for lenvatinib and 3.7 months (0.1–38.7) for sorafenib. Discontinuation due to adverse events occurred in 13% of lenvatinib-treated and 9% of sorafenib-treated patients.

The analysis of secondary end points brought some elements worth of discussion. In particular, the discrepancy between the stark superiority of lenvatinib in terms of TTP (7.4 vs 3.7 months) and objective response rate (24 vs 9%) on one hand, and the lack of a full superiority in terms of OS raises further doubts about the well-known limits of surrogate end points in the evaluation of systemic treatments for HCC. However, at the time of writing this review, results of the REFLECT trial are available only as conference presentation, major considerations will be therefore feasible only after publication of full data.

In conclusion, the successful REFLECT trial will lead to the availability of a new drug for the treatment of advanced HCC. Even if lenvatinib failed to reach a full superiority compared with sorafenib, the competition between companies may lead to more affordable costs of anti-angiogenic drugs for lower-income countries and therefore to a more widespread opportunity of care. However, once lenvatinib will be approved for treating HCC patients, criteria for the differential use of sorafenib and lenvatinib as first-line therapy will need to be established.

### Regorafenib

Regorafenib is an oral multikinase inhibitor with a structure very similar to that of sorafenib except for the addition of a fluorine atom in the center phenyl ring which confers a distinct biochemical and pharmacologic profile [64]. Its molecular targets include VEGFR-2, VEGFR-3, Tie-2, PDGFR, FGFR-1 and the mutant oncogenic kinases KIT, RET and B-RAF [65].

Tie-2 is predominantly expressed on endothelial cells and is a crucial regulator of angiogenesis, indispensible for the maturation of immature vessels. Thus, thanks to the combined blockage of VEGFR2 and Tie-2 signaling, regorafenib potentially inhibits tumor neoangiogenesis more potently than agents blocking VEGF signaling alone [66].

Regorafenib is the first agent that has been shown a significant survival benefit when administered as second-line therapy after sorafenib failure. In the randomized, placebo-controlled Phase III international study (RESORCE trial), regorafenib significantly improved OS compared with placebo in HCC patients who had experienced radiologic progression during sorafenib therapy (HR: 0.63; 95% CI: 0.50, 0.79; p < 0.0001) [67].

The RESORCE study was conducted at 152 centers in 21 countries, in North America, South America, Europe, Asia and Australia. The patients who were eligible for the trial had progressed under sorafenib and were able to tolerate at least 400 mg of sorafenib daily for at least 20 of the last 28 days prior discontinuation. Patients were randomized 2:1 to supportive care plus either regorafenib 160 mg once-daily or placebo. Treatment consisted of cycles of 3 weeks on the agent followed by a 1-week break and was continued until disease progression, death, or unacceptable toxicity. Patients had to be randomized within 10 weeks of their last sorafenib dose. After stratification for various factors, including age, extrahepatic disease, macrovascular invasion and underlying disease, patients treated with regorafenib still had a significantly better OS [11].

The safety profile was generally consistent with that reported for other gastrointestinal malignancies [68]. In particular, rates of grade ≥3 adverse events were 79.7% with regorafenib and 58.5% with placebo. Most common grade ≥3 adverse events included hypertension (15.2 vs 4.7%), hand–foot skin reaction (12.6 vs 0.5%), fatigue (9.1 vs 4.7%) and diarrhea (3.2 vs 0%). Rates of interruptions or dose modifications due to adverse events were 68% with regorafenib and 31% with placebo, while 25% of patients in the regorafenib group versus 19% of patients in the placebo group discontinued due to adverse events.

In a *post hoc* exploratory analysis, the occurrence of hand–foot skin reaction during treatment suggested that this adverse event may be a marker for drug activity. Overall survival in patients developing hand–foot skin reaction was significantly longer compared with those who did not experience it (14.1 vs 6.6 months; HR: 0.52; 95% CI: 0.40–0.67), as was previously shown for sorafenib. [69]

Interestingly, in the updated analysis of the RESORCE trial, the assessment of outcomes for the treatment sequence of sorafenib plus regorafenib showed an OS of 26 months from the start of sorafenib treatment among patients who received the sorafenib/regorafenib sequential therapy (19.2 months in the placebo group) [70]. This important extension in OS suggests that sequential systemic treatments may further improve the prognosis of a subgroup of patients with advanced HCC who are likely characterized by an appropriate management of adverse events during first-line sorafenib therapy and adequate selection for second-line treatment.

The 26 month OS observed with the sequential sorafenib/regorafenib therapy is in fact comparable with the conventional prognosis of patients with intermediate stage after TACE [49].

#### Cabozantinib

Cabozantinib is an oral multikinase inhibitor targeting MET in addition to VEGFR2. It also inhibits many other receptor tyrosine kinases (such as RET, KIT, AXL and FLT3) that have been implicated in neoplastic pathobiology [71]. Cabozantinib is currently approved by the US FDA for the treatment of advanced renal cell carcinoma and of medullary thyroid cancer.

The rationale for a dual VEGFR/MET blockade is supported by the consolidated evidence that resistance to VEGFR-targeted therapies may arise from the upregulation of alternative proangiogenic and proinvasive signaling pathways, including the MET pathway [72–74].

These aspects have been confirmed in the specific case of HCC as well, as the rate of MET-high HCC increased from a pre-sorafenib value of 40% to an almost double value after progression to sorafenib in a biomarker study of the eventually failed tivantinib trial [75].

A role of cabozantinib has been investigated in a Phase II randomized-discontinuation study in advanced HCC patients who had received one prior therapy, showing promising results and an overall disease control rate of 68%. The most common grade 3/4 adverse events were diarrhea (17%), palmar–planter erythrodysesthesia (15%) and thrombocytopenia (10%) [76].

Based on these data, the CELESTIAL Phase III trial (NCT01908426) compared cabozantinib with placebo in patients with HCC and Child-Pugh A liver function who received prior sorafenib and may have received up to two prior systemic cancer therapies, making it a second- to third-line clinical trial [77].

The enrollment was completed in September 2017 and on October 2017 Exelixis announced that the CELESTIAL trial met its primary end point showing a statistically significant and clinically meaningful improvement in OS of patients treated with cabozantinib compared with placebo in advanced HCC [78].

## Ongoing Phase III trials

### Ramucirumab

Ramucirumab is a recombinant IgG1 monoclonal antibody antagonizing and the VEGFR-2 [5]. The original Phase III REACH trial investigated the efficacy of the safety of this molecule as a second-line treatment for unresectable HCC. The most common grade 1 or 2 treatment-emergent adverse events were peripheral edema (36%), ascites (22%) and headache (18%). Any grade events that occurred at a higher frequency in the ramucirumab group than the placebo group included bleeding or hemorrhage (32%), which was primarily due to grade 1–2 epistaxis (14%) and gingival bleeding (6%); hypertension (20%), proteinuria (17%), liver injury or failure (51%), and infusion-related reactions (7%).

No benefit in OS was observed in the ramucirumab treatment arm. However, the survival analyses of patient with baseline alpha-fetoprotein (AFP) concentration ≥400 ng/ml (which had been predefined in the study plan) shows a meaningful effect of the study drug on OS (HR: 0.674; median OS: 7.8 vs 4.2 months; p = 0.006) [5].

Unfortunately, no stratification according to AFP values at the time of inclusion had been included in the original design of the study. As such, the novel Phase III REACH-2 study (NCT02435433) will compare ramucirumab versus placebo in patients with AFP >400 ng/ml [79].

The enrollment is currently closed and results are expected in late 2018 or 2019.

## Immune checkpoint inhibitors

The escape from immunological surveillance is one of the main mechanisms of tumor progression. It can be the consequence of distinct immune alterations, including defective antigen presentation, dysfunctions of effector and regulatory T cell, alterations in immune checkpoint molecules, disarray of cytokine profiles. Their identification has provided the main rationale for the development of immunotherapy in HCC [80].

Outstanding achievements with immune checkpoint inhibitors in different malignancies have been reported in the last years. The best known checkpoint inhibitors are nivolumab, an inhibitor of the lymphocytes PD1 receptor, and ipilimumab, an inhibitor of CTLA-4. Also, a different PD1 inhibitor (pembrolizumab) and the antagonists of PD-L1 durvalumab, avelumab and atezolizumab have been developed [81]. All of these drugs inhibit an immune checkpoint exploited by the tumor cells to protect themselves from the immune system recognition and destruction [81,82]. As a matter of fact, nivolumab and ipilimumab revolutionized the treatment of metastatic melanoma and non-small-cell lung carcinoma.

The first evidences supporting the rationale of immune checkpoint inhibitors in HCC came from an investigator-initiated Phase II clinical trial of the CTLA-4 inhibitor tremelimumab [83]. In this study, Sangro and collaborators recruited 21 HCV-related HCC, 57% in the advanced stage, 42.9% with Child-Pugh class B and 23.8% previously treated with sorafenib. Patients were treated with tremelimumab until progression or unacceptable toxicity. Partial response rate and disease control rate were 17.6 and 76.4%, respectively. Median TTP was 6.48 months (95% CI: 3.95–9.14). A significant drop in viral load was observed and this antiviral effect was associated with an enhanced specific anti-HCV immune response.

Even if some transient grade 3–4 aminotransferase elevation developed, no toxicities requiring systemic steroid treatment were registered.

Recently, the effects of tremelimumab have been studied in combination with locoregional therapy in 32 HCC patients, with the aim to enhance the tremelimumab activity by inducing immunogenic tumor cell death and local Inflammation. Tremelimumab was administered with transarterial chemoembolization in seven patients with BCLC-B, with radiofrequency ablation in ten patients with BCLC-C, and in 11 patients with BCLC-C underwent cryoablation. Tumor response was assessed in the 19 patients with evaluable lesions outside of the ablation area and the partial response rate was 26% (5/19), while the disease control rate was 84% (16/19) [84].

Subsequently, a large Phase IB/II multicohort trial investigating the safety of nivolumab alone or associated with ipilimumab was announced and is currently ongoing [85].

As of August 2017, the CheckMate-040 study (NCT01658878) includes: two dose escalation/expansion cohorts (cohort 1 and 2); a cohort for a randomized 1:1 comparison versus sorafenib in the frontline setting (cohort 3); a combination therapy cohort with ipilimumab for patients progressing or intolerant to sorafenib (cohort 4); a cohort reserved to patients with Child-Pugh B HCC (cohort 5); and a combination therapy cohort with cabozantinib (with or without ipilimumab; cohort 6) [85,86].

Results from the first two cohorts are now available and encouraging. In a total sample of 262 patients, grade 3/4 treatment-related adverse events occurred in 20%. The objective response rate (ORR) was 20% (95% CI: 15–26) in 214 patients treated in the dose-expansion phase with a median duration of response of 9.9 months and a disease control rate of 64% (95% CI: 58–71) [87].

Results from cohort 3, 4 and 5 are pending (closed enrollment), while cohort 6 is still active and recruiting both sorafenib-naive and pretreated patients.

These preliminary results were convincing enough to promote the global Phase III RCT of nivolumab versus sorafenib as first-line treatment in patients with advanced HCC (CheckMate-459: NCT02576509) [88].

In parallel, another Phase III RCT is comparing pembrolizumab versus best supportive care in a second-line setting [89].

## Metronomic capecitabine

Metronomic regimens are progressively gaining interest and popularity in oncology. Metronomic protocols rely on the continuous administration of low doses of antineoplastic drugs, without prolonged drug-free breaks. The potential efficacy of metronomic chemotherapy depends on multiple factors, including: reduction of the therapeutic resistance of the tumor, inhibition of tumoral angiogenesis and activation of both innate and adaptive immune response [90,91].

Until now, cytotoxic drugs administered according to standard protocols did not demonstrate a clear OS benefit in HCC patients [1]. Data about metronomic regimens, however, are still scarce. Metronomic capecitabine (MC) was evaluated in three different studies, mainly in a second-line setting. All of these studies showed a low rate of adverse events and a good efficacy [92–95]. However, the lack of prospective RCTs does not allow a definite judgement of MC biological efficacy.

Such studies would require a large amount of independent funds, as capecitabine patent has expired. Answering these calls, the Italian Medicines Agency recently financed a cost–effectiveness, randomized, cross-over study comparing capecitabine versus sorafenib [96]. This trial will include multiple Italian centers and will provide interesting information about MC both in frontline and in second-line setting.

## Conclusion & future perspective

Systemic treatment of HCC is rapidly evolving after a decade of disappointment and, at present, three new drugs (i.e., regorafenib, lenvatinib, cabozantinib) have been shown to be effective in Phase III clinical trials (Table 3).

**Table 3.** **Phase III randomized, controlled, clinical trials who met primary end point for patients with unresectable hepatocellular carcinoma.**

**Drug**	**Study (year)**	**Line**	**Study design**	**Median OS months (95% CI)**	**HR (95% CI)**
Sorafenib	SHARP (2006)	1	Randomized, placebo-controlled	10.7 (9.4–13.3) vs 7.9 (6.8–9.1)	0.69 (0.55–0.87)

	Asia-Pacific (2007)	1	Randomized, placebo-controlled	6.5 (5.56–7.56) vs 4.2 (3.75–5.46)	0.68 (0.50–0.93)

Regorafenib	RESORCE (2016)	2	Randomized, placebo-controlled	10.6 (9.1–12.1) vs 7.8 (6.3–8.8)	0·63 (0.50–0.79)

Lenvatinib	REFLECT (2017)	1	Randomized, non-inferiority, lenvatinib vs sorafenib	13.6 (12.1–14.9) vs 12.3 (10.4–13.9)	0.92 (0.79–1.06)

Cabozantinib	CELESTIAL (2017)	2–3	Randomized, placebo-controlled	Publication of data is expected in 2018	

HR: Hazard ratio; OS: Overall survival.

Sorafenib remains the standard-of-reference in HCC systemic therapy, as of today. This role derives from the lack of clearly superior drugs as first-line treatment, and from the wide availability of real-life clinical data. It is entirely possible that lenvatinib may become a competitor frontline therapy; however, due to the similar mechanism of action and toxicity profile, it is more likely that in the next future its role in the first-line setting will be challenged by immune checkpoint inhibitors or their combination.

In the second-line scenario, the treatment sequence of sorafenib plus regorafenib is associated to an important extension in overall survival making the prognosis of this subgroup of patients with advanced HCC similar to that of those with intermediate stage underwent TACE. After positive results of the CELESTIAL trial, cabozantinib could become a further therapeutic alternative for patients failing sorafenib, although, at present we do not know the potential benefit of the sequence of sorafenib/cabozantinib.

Over the next 5 years, the scenario will depend on the success or failure of the immunotherapy trials. If successful, these new drugs might have a relevant role both in the first- and second-line setting as they could be the optimal strategy for patients with contraindication or poor tolerability to multikinase inhibitors. Moreover, if confirmed that immunotherapy can be carried out independently of causes less liver toxicity, this approach could allow treatment of HCC even in patients with more compromised liver function.

Immunotherapy could also potentially extend its therapeutic indication to the adjuvant setting, given the promising preliminary results in this field (where, on the contrary, sorafenib has shown no benefit). However, all of these potential uses are subject to ongoing or future trials in order to demonstrate a survival benefit.

Finally, the identification of biomarkers that could predict response or resistance to treatments is ideally the best way to guide treatment decision and to identify the best timing for a therapy switch. For this purpose, collection of tumor histological samples and biobanking in clinical trials is essential.

**Executive summary**

- To date, sorafenib is the only systemic agent globally available for use in patients with hepatocellular carcinoma (HCC).
- The efficacy and safety of sorafenib were assessed in two large double-blind, placebo-controlled, Phase III randomized-controlled trials and confirmed in large real-life clinical trials.
- No biomarkers predictive of response to sorefenib are available. However, patients suffering from early dermatological adverse events seem to have a better prognosis.
- Sorafenib treatment may be of benefit even after radiological progression. With second-line treatments available at the horizon, future studies should identify the more appropriate timing for switching therapies.
- In the Phase III REFLECT trial, lenvatinib has shown to be non-inferior to sorafenib in a front-line setting.
- In the second-line setting, both regorafenib and cabozantinib have shown to be superior to best supportive care following sorafenib failure. The treatment sequence of sorafenib plus regorafenib showed an OS of 26 months in patients who received the sorafenib/regorafenib sequential therapy.
- Immune checkpoint inhibitors are attracting more and more attention, especially after interesting preliminary results. Nivolumab is being tested in a frontline setting versus sorafenib. At the same time, tremelimumab is under investigation in a second-line trial versus best supportive care.
- The potential role of immunotherapy in patients who cannot tolerate multikinase inhibitors, in those with more deteriorated liver function, and in the adjuvant setting makes this therapeutic approach of particular interest in HCC patients.
